# *Lactococcus lactis* Subsp. *lactis* LL-1 and *Lacticaseibacillus paracasei* LP-16 Influence the Gut Microbiota and Metabolites for Anti-Obesity and Hypolipidemic Effects in Mice

**DOI:** 10.3390/antiox14050547

**Published:** 2025-05-01

**Authors:** Peng Gao, Yuanyang Nie, Lili Zhao, Jing Zhang, Wupeng Ge

**Affiliations:** 1 College of Food Science and Engineering, Northwest A&F University, Xianyang 712100, China; gaopeng221@nwafu.edu.cn (P.G.); lili.zhao@nwsuaf.edu.cn (L.Z.); jingzhang@nwafu.edu.cn (J.Z.); 2 School of Food Science, Henan Institute of Science and Technology, Xinxiang 453003, China; yuanyang8238@hist.edu.cn

**Keywords:** lactic acid bacteria, oxidative stress, short-chain fatty acids, gut microbiota, metabolomics

## Abstract

This study utilized a high-fat diet-induced obese male C57BL/6 mice model to investigate the anti-obesity and lipid-lowering effects of *Lactococcus lactis* subsp. *lactis* LL-1 and *Lacticaseibacillus paracasei* LP-16. A gut microbiota analysis via 16S rRNA sequencing, along with measurements of body weight, lipids, inflammation markers, and gut metabolites, revealed that lactic acid bacteria (LAB) significantly reduced body weight, blood lipid levels, and liver oxidative stress. They also enhanced gut microbiota diversity and evenness, potentially by modulating the *Firmicutes*/*Bacteroidetes* ratio to limit excess energy absorption. Malondialdehyde (MDA) showed extremely significant positive correlations with *Lachnospiraceae*, *Blautia*, and *Colidextribacter*, and a significant positive correlation with *Helicobacter,* while superoxide dismutase (SOD) and glutathione peroxidase (GSH-Px) exhibited opposite trends. Specifically, *Muribaculaceae*, *Bacteroides*, and *Lactobacillus* showed negative correlations with MDA levels and positive correlations with SOD and GSH-Px. Short-chain fatty acids (SCFAs) positively correlated with *Muribaculaceae*, *Bacteroides*, *Mucispirillum*, and *Lactobacillus,* but negatively correlated with *Lachnospiraceae*, *Blautia*, *Colidextribacter*, *Alistipes*, and *Helicobacter*. They increased SCFA levels by promoting beneficial bacteria and reducing pathogens, alleviating obesity and hyperlipidemia. Additionally, they regulated the gut microbiota, decreasing bile acids and long-chain fatty acids while increasing SCFAs, short peptides, and vitamins, thereby improving gut metabolic disorders and enhancing host gut health.

## 1. Introduction

There are many kinds of bacteria in the human gastrogut tract, and with 10 times more microbial cells than human cells, these microorganisms affect human health by biological antagonism and effects on the immune system and regulation of metabolism [1]. The gut flora of healthy people includes *Bacteroidetes*, *Actinobacteria*, *Firmicutes*, *Proteobacteria*, *Verrucomicrobiota*, and *Clostridium*, with only small amounts of other types of bacteria, viruses, and eukaryotes [2]. *Bacteroidetes* and *Firmicutes* account for about 90% of the gut flora [3]. Gut microbes are essential determinants of host physiological health. An imbalance of the flora will affect the metabolism and transformation of nutrients such as sugar, fat, and protein, thus endangering human health and causing diseases such as metabolic diseases (obesity, hyperlipidemic disease, hyperglycemia, and hypertension), psychiatric diseases (anxiety, depression, and autism) and liver diseases (fatty liver and non-alcoholic hepatitis).

Gut bacteria help regulate and maintain human lipid metabolism, which can affect energy balance, promote fat storage, and cause lipid metabolism disorders. When the microflora of normal mice were transplanted into sterile mice, the energy storage of sterile mice was increased, and their fat content and liver triacylglycerol content were increased, indicating that gut bacteria can supplement energy intake. Gao et al. built a high-fat mouse model and compared mice in the normal group and the high-fat group [4]. The content of *Bacteroides* increased significantly after one week of high-fat feeding, and the ratio of *Firmicutes* to *Bacteroides* changed from 0.86 to 1.77 after eight weeks of feeding, while the content of *Verrucomicrobiota* in the high-fat group ascended significantly and *Escherichia coli* decreased significantly.

Probiotics, which mainly include *Lactobacillus* and *Bifidobacterium*, are defined as microorganisms that are beneficial to the host. Probiotics can regulate gut flora disorder and improve hyperlipidemia. Nakamura et al. administered *Amylolactobacillus amylophilus* to 116 obese mouse models and found significantly reduced levels of low-density lipoprotein (LDL-C) and TG in serum and significantly increased levels of high-density lipoprotein (HDL-C) [5]. Guardamagna et al. tested 38 children with dyslipidemia and found that oral *Bifidobacterium* probiotics for the treatment of primary hyperlipidemia were well tolerated and had a significant effect [6]. A meta-analysis report showed that probiotic intake affected the levels of high-density lipoprotein and serum cholesterol, which indirectly reduced blood lipids [7]. Thus, the establishment of normal gut flora could balance lipid metabolism. *Bifidobacterium* can also reduce the concentration of blood lipids and the symptoms of metabolic disorders such as obesity [8]. After treatment with *Lactobacillus*, the levels of low-density lipoprotein, triglyceride, and total cholesterol in the body, and triglyceride and total cholesterol in the liver were significantly reduced.

By supplementing with probiotics and probiotic fermented foods, the structure and diversity of gut flora can be significantly improved, and the microbial changes can help the treatment and prevention of hyperlipidemia. Some lactic acid bacteria (LAB) isolated and identified from traditional fermented foods have excellent probiotic properties, suggesting these foods are important sources of functional probiotics. In this study, two independently isolated LAB strains were tested for the treatment of mice with metabolic syndrome induced by a high-fat diet. The weight, lipid, inflammation index, gut microbiota, and metabolites of the mice were measured and used as indicators to evaluate the ability of these strains to improve metabolic syndrome.

## 2. Materials and Methods

### 2.1. LAB Strains and Culture Conditions

Lyophilized strains of *Lactococcus lactis* subsp. *lactis* LL-1 (LL) and *Lacticaseibacillus paracasei* LP-16 (LP) were isolated from traditional fermented dairy products in the western pastoral region of China and are preserved in Microbial Culture Collection Center of the College of Food Science and Engineering, Northwest A&F University (Yangling, Xianyang, Shaanxi, China) with the accession number QH-SMN-07-3-L and GY-L004, respectively. These two LAB are on the list of food-safe bacteria published by the China National Center for Food Safety Risk Assessment. The LAB were cultured in Man–Rogosa–Sharpe (MRS) broth (Guangdong Huankai Microbial Technology Co., Ltd., Guangzhou, China) for 48 h at 37 °C, then centrifuge at 4 °C and 4000 r/min for 10 min to collect the microbial biomass, and resuspend in sterile water to obtain a LAB suspension (the final concentration of LAB was ×10^8^ CFU/mL).

### 2.2. Animals, Diets, and Experimental Design

C57BL/6 male mice aged 6 weeks (*n* = 60) were obtained from the Experimental Animal Center of Xi’an Jiaotong University (Xi’an, China). The determination of sample size was based on the number of experimental groups and the need to minimize experimental errors arising from individual differences. Experiments were carried out following the Guide for the Care and Use of Laboratory Animals [9] and Measures for the Management of Laboratory Animals of Northwest A & F University. The study protocol was approved by the Animal Care and Use Committee, Northwest A & F University (Yangling, Shaanxi, China) (approval no. IACUC2024-0586, date of approval: 27 June 2024).

The mice in each group were housed under conditions of 25 ± 1 °C, (60 ± 5)% relative humidity, and a 12/12 h light–dark cycle, with free access to the standard diet (SD), water, and movement, and acclimated for one week before initiating the experiment. After one week of the adaptation period, the mice were randomly divided into six groups of 10 mice each (two cages for each group, and 5 mice for each cage). Random numbers were generated using the standard = RAND() function in Microsoft Excel. The grouping and gavage conditions of the mice were as follows: (1) control group (C)—the mice were fed the standard diet (AIN-93M) and orally administered 200 μL normal saline; (2) C+LL group—the mice were fed the standard diet (AIN-93M) and orally administered 200 μL LL suspension; (3) C+LP group—mice were fed the standard diet (AIN-93M) and orally administered 200 μL LP suspension; (4) high-fat diet group (HFD)—the mice were fed the high-fat diet (TP23100, 4.5 kcal/g heat) and orally administered 200 μL normal saline; (5) HFD+LL group—the mice were fed the high-fat diet (TP23100, 4.5 kcal/g heat) and orally administered 200 μL LL suspension; (6) HFD+LP group—the mice were fed the high-fat diet (TP23100, 4.5 kcal/g heat) and orally administered 200 μL LP suspension. The sample size and oral gavage dosage of LAB were determined with reference to the in vivo hypolipidemic study of *Lactobacillus casei* YBJ02 by Qian et al. [10]. The mice in each group were orally administered once a day at am 9:00–11:00 for 8 weeks, and the gavage order was randomized daily, with each mouse receiving the treatment at different times each day. The body weight of the mice was measured and recorded weekly during the period of gavage. On the final day after the intervention with LAB, fasting was implemented for 12 h without water. At am 9:00 of the next day, blood was collected from the eyeball and placed in a non-enzymatic tube, and then all the mice were sacrificed by cervical dislocation, and the colons and ceca were aseptically removed to cryogenic vials and immediately saved in liquid nitrogen.

This study did not establish a disease model. Instead, gastric gavage was administered to each group of mice from the outset of the experiment. Upon the completion of the experiment, comparative analyses were conducted on various serum biochemical indicators, hepatic oxidative stress levels, gut microbiota, and metabolites. Consequently, no specific criteria were applied for the inclusion or exclusion of mice. Each mouse was handled by three different researchers: the first researcher performed the procedures according to the randomization table. This individual was the only one aware of the experimental group assignments. The second researcher was responsible for the oral gavage administration. Finally, a third researcher, who was blinded to both group allocation and experimental treatments, measured and evaluated the experimental outcomes. If oral gavage may lead to adverse consequences such as feeding difficulties, reduced food intake, and weight loss in mice, we will implement monitoring through twice-daily observations, biweekly weighing, and the temporary suspension of gavage as nursing measures. Should the symptoms persist after nursing interventions, humane endpoint euthanasia will be performed on the animals. Fortunately, no adverse events were observed in any of the mice during the study period, and at the conclusion of the gavage experiment, all the mice survived normally (n = 10 in each group).

### 2.3. Determination of Serum Biochemical Indices, Oxidative Stress Level, and Hematoxylin–Eosin Staining of Mice Liver

After sacrificing the mice, the livers of the mice were taken to wash with physiological saline. After absorbing the water, they were weighed. The formula for calculating the liver index is as follows: Liver index (%) = liver mass (g)/body mass (g) × 100%. The concentrations of total cholesterol (TC), triglyceride (TG), high-density lipoprotein cholesterol (HDL-C), and low-density lipoprotein cholesterol (LDL-C), and the activity of aspartate transaminase (AST) and alanine aminotransferase (ALT) in the serum of the mice, malondialdehyde (MDA), superoxide dismutase (SOD), and glutathione peroxidase (GSH-Px) in the liver of the mice were detected using kits provided by Nanjing Jiancheng Bioengineering Research Institute Co., Ltd. (Nanjing, China) according to the kit instructions. Hematoxylin–eosin staining follows the method of Manne et al. [11].

### 2.4. Determination of SCFAs

The extraction and determination of short-chain fatty acids (SCFAs) in feces was performed as described by Qu et al. [12]. Briefly, a 0.1000 g fecal sample was weighed in a 10 mL centrifuge tube, 1 mL ultra-pure water was added, and the sample was swirled thoroughly for 10 min. Next, 0.15 mL 50% H_2_SO_4_ solution was added and mixed, 1.6 mL anhydrous ether was added and immediately mixed, and then the mixture was incubated at 4 °C for 20 min, reversing twice. After centrifugation at 12,000 r/min for 5 min, 1 mL of the upper ether liquid into a concentration tube was removed and then nitrogen was gently blown to concentrate the solution to half of the original volume. After performing this process on ice, it was filtered and then transferred to the test bottle to be measured. Measurement was performed using a GC Instrument: Shimadzu 2014C; Carrier gas: N2; Shunt ratio: 10:1; Flow speed: 2.0 mL/min; Column: DB-FFAP; Temperature rise procedure: maintain at 50 °C for 1.0 min, rise to 120 °C at 15 °C/min, rise to 170 °C at 5 °C/min, rise to 220 °C at 15 °C/min for 5 min, sample temperature: 250 °C; Detector: hydrogen flame detector FID; temperature of the detector: 270 °C.

### 2.5. Genomic DNA Extraction and 16S rRNA High Throughput Sequencing

Total genomic DNA was extracted by QIAamp DNA Stool Mini Kits (Qiagen, Duesseldorf, Germany) from mouse gut contents. For each sample, the sequencing and bioinformatics analysis was carried out by Novo-gene Co., Ltd. (Beijing, China). The resulting DNA was identified by agarose electrophoresis and quality was assessed by NanoDrop (Thermo Fisher Scientific, Waltham, MA, USA). High-fidelity PCR was used to amplify the 16S rRNA gene V3 + V4 region using Primer 341F (5′-CCTAYGGGRBGCASCAG-3′) and Primer 806R (5′-GGACTACNNGGGTATCTAAT-3′). The PCR system included the following: 10 μL template DNA, 2 μL upstream and downstream primers, 15 μL Phusion High-Fidelity PCR Master Mix (New England Biolabs, Beverly, MA, USA), and 21 μL ddH_2_O. The amplification conditions were as follows: predenaturation at 98 °C for 1 min; 98 °C, 10 s, 50 °C, 30 s, 72 °C, 30 s, for 30 cycles; at last, 72 °C, extended for 5 min, with ddH_2_O used as a template for negative control. The PCR products were detected by 2% agarose gel electrophoresis, and a TruSeq DNA PCR-Free Sample Preparation Kit library construction Kit (Illumina, San Diego, CA, USA) was used for library construction. The constructed library was quantified by a Qubit 2.0 Fluorometer (Thermo Fisher Scientific, Waltham, MA, USA) and Agilent BioAnalyzer 2100 system (Agilent Technologies, Santa Clara, CA, USA), and then sequenced on the Illumina NovaSeq 6000 platform (Illumina, San Diego, CA, USA) to generate 2 × 250 bp paired-end reads.

The paired-end reads were merged using FLASH (V1.2.11, http://ccb.jhu.edu/software/FLASH/ (accessed on 21 october 2024)). Quality filtering on the raw tags was performed under specific filtering conditions to obtain high-quality clean tags according to the QIIME (V1.9.1, http://qiime.org/scripts/split_libraries_fastq.html (accessed on 21 october 2024)) quality-controlled process. Sequence analyses were performed using the Uparse software (Uparse V7.0.1001, http://drive5.com/uparse/ (accessed on 21 october 2024)) Sequences with ≥97% similarity were assigned to the same OTUs. A representative sequence for each OTU was screened for further annotation. For each representative sequence, the SSUrRNA Database of Silva132 (http://www.arb-silva.de/ (accessed on 21 october 2024)) was used based on the Mothur algorithm to annotate taxonomic information.

### 2.6. Metabolomic Analysis of Gut Contents in Mice

A 60 mg sample of gut content was weighed and placed into a 1.5 mL centrifuge tube. Two small steel beads and 600 μL of a methanol–water solution (*v*:*v* = 4:1, containing a mixed internal standard at 4 μg/mL) were added. The mixture was precooled in a −40 °C refrigerator for 2 min, then homogenized using a grinder at 60 Hz for 2 min. The sample was subsequently extracted in an ice-water bath with ultrasonic treatment for 10 min and left to stand at −40 °C overnight. After centrifugation at 4 °C and 12,000 rpm for 10 min, 150 μL of the supernatant was collected using a syringe, filtered through a 0.22 μm organic phase pinhole filter, and transferred to an LC injection vial. The sample was stored at −80 °C until an LC-MS analysis. The LC-MS detection of gut metabolites, along with the preprocessing and statistical analysis of metabolomics data, was conducted by Shanghai OE Biotech Co., Ltd. (Shanghai, China).

### 2.7. Statistical Analysis

The experimental data are presented as mean ± standard deviation. Statistical analysis of significant differences between experimental groups was performed using the SPSS Statistics software (V27.0.1, IBM Corporation, Armonk, NY, USA), with post hoc multiple comparisons conducted using Duncan’s test. A *p*-value < 0.05 was considered statistically significant. The diversity of the gut microbiota within individual mouse samples (α-diversity) was evaluated using the chao1, observed species, Shannon, and Simpson indices, which were computed with the QIIME software (V1.9.1). To determine significant differences in microbial community composition between the control and treatment groups (β-diversity), permutational MANOVA (pMANOVA) based on weighted UniFrac distances (Bray–Curtis distance method) was employed, and the results were visualized through principal coordinates analysis (PCoA). Comparisons of α-diversity and β-diversity between the control and treatment groups were conducted using the Tukey test and Wilcoxon rank-sum test in the R software (V4.4.3, https://www.r-project.org/). Additionally, statistical analysis of species-level differences between the control and treatment groups was performed using a permutation test in the R software (V4.4.3, https://www.r-project.org/).

## 3. Results and Discussion

### 3.1. Changes in Body Mass in Mice During Feeding Period

**Figure 1:**
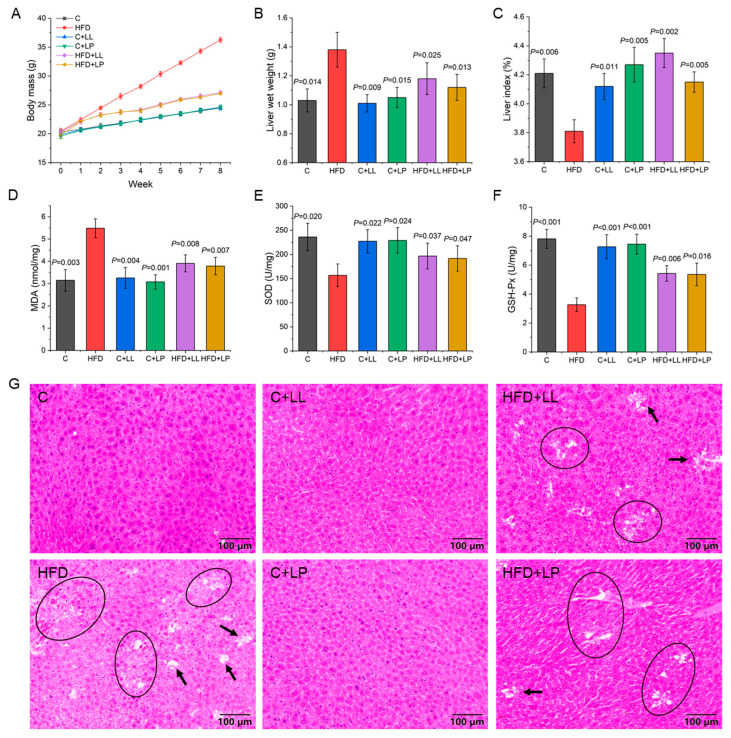
Effects of lactic acid bacteria on liver indices and oxidative stress level in mice: (**A**) body mass; (**B**) liver wet weight; (**C**) liver index; (**D**) MDA; (**E**) SOD; (**F**) GSH-Px; (**G**) pathological sections of mice liver by hematoxylin–eosin staining. The black arrow and circle indicate fat granules. Compared with the HFD group, the difference was statistically significant at *p* < 0.05.

The average body weights of each group of mice during the 8-week experiment were compared, and the results are shown in Figure 1A. At week 0, the average initial body mass of the model mice was 20.0 g. As the experiment progressed, the body mass of the mice exhibited a consistent upward trend across all the experimental groups. The success criterion of the C57BL/6J obese mouse model is an average body mass of the high-fat diet (HFD) group 20% higher than that of the control group. The data presented in Figure 1A reveal that 8 weeks of high-fat diet feeding resulted in significantly elevated body mass in the HFD group (148% of control values, *p* < 0.05), thereby validating the successful induction of the obesity model. After LAB treatment, compared with the HFD group, the body mass of the mice in the intervention group showed different degrees of decline. From the 3rd week, the average body mass of mice in the HFD+LL and HFD+LP groups was significantly lower than that of the mice in the HFD group (*p* < 0.05). There was no significant difference in body mass between the C+LL, C+LP, and the control group throughout the intervention period.

### 3.2. Serum Biochemical Indices of Mice

As presented in Table 1, significant metabolic alterations were observed in the HFD group, including increased TC, TG, and LDL-C levels coupled with decreased HDL-C concentrations (all *p* < 0.05) relative to the control group. Obese mice showed hyperlipidemia. The AST and ALT activities of the mice in the HFD group were also significantly increased, reaching (188.90 ± 8.58) U/L and (110.49 ± 6.38) U/L, respectively, indicating that a long-term high-fat diet caused certain damage to the liver of the mice. Compared to the HFD group, LL and LP treatment resulted in significant lipid profile improvements, including reduced TC, TG, and LDL-C levels (*p* < 0.05) and elevated HDL-C concentrations (*p* < 0.05). Importantly, TG and LDL-C levels were effectively normalized in both treatment groups. The activities of ALT and AST also decreased significantly under LL and LP, to (154.10 ± 8.85) U/L and (70.91 ± 5.66) U/L, respectively, in the HFD+LL group, and to (151.55 ± 8.62) U/L and (71.98 ± 5.89) U/L, respectively, in the HFD+LP group. These results show that the intake of LL and LP can significantly reduce the level of lipids in obese mice and somewhat alleviate the liver damage caused by a long-term high-fat diet. LAB showed no significant effect on the serum biochemical indices in normal mice during the whole intervention period.

For a person with obesity, non-esterified fatty acids will be released into the circulatory system and circulate in the body, thereby increasing TG level, reducing HDL-C level, transforming lipid composition to LDL-C, and increasing the risk of atherosclerosis [13]. The lipid-lowering effects of probiotics and prebiotics have been well documented in clinical studies, showing significant reductions in TC and LDL-C levels among overweight and obese subjects [14]. These findings support the hypothesis that LAB plays a crucial role in lipid metabolism regulation. The formation of fatty liver disease is related to the enhancement of inflammatory response, and AST and ALT activities are significantly increased in the early stage of fatty liver formation [15]. Some *Lactobacillus* can significantly inhibit the development of obesity and lipid deposition in mice by reducing TG levels and serum AST and ALT activities [16]. LAB intervention effectively alleviates the liver damage of HFD mice and plays an important role in preventing fatty liver and liver lesions.

### 3.3. Liver Indices and Oxidative Stress Level of Mice

The high-fat diet (HFD)-induced obesity model demonstrated significant hepatic alterations beyond increased body weight, including elevated liver wet weight and reduced liver index. Compared to the control animals, the HFD-fed mice exhibited a 33.98% increase in liver wet weight (*p* < 0.05, Figure 1B) and a 0.4% decrease in the liver index (*p* < 0.05, Figure 1C), confirming successful obesity induction and substantial hepatic impact. LAB intervention effectively mitigated these changes, with the HFD+LL and HFD+LP groups showing 14.49% and 18.84% reductions in liver wet weight, respectively (*p* < 0.05, Figure 1B), demonstrating LAB’s therapeutic potential in ameliorating obesity-related hepatic alterations. After LAB intervention, the liver index of the mice was significantly increased (*p* < 0.05) and recovered to the control level (Figure 1C), indicating that the liver index of obese mice could be significantly increased by the gavage of LAB. In addition, there were no significant differences in liver wet weight and liver index between the C+LL, C+LP group, and control group, indicating that LAB had no significant effects on the liver wet weight and liver index of normal mice. Li et al. utilized soybean milk fermented by *Lactiplantibacillus plantarum* HFY01 to reduce weight and fat in HFD-induced obese mice [17]. The results indicated that the fermented soybean milk could lower the body fat percentage and liver index of the obese mice, and effectively inhibit obesity induced by a high-fat diet, demonstrating significant potential for application.

Oxidative stress represents a pathological state characterized by disrupted homeostasis between pro-oxidant and anti-oxidant systems, primarily resulting from the excessive accumulation of reactive oxygen species (ROS) and impaired cellular defense mechanisms. MDA content, SOD, and GSH-Px activity can reflect the degree of lipid peroxidation to some extent [18]. Compared with the control group, the MDA content of the HFD group was significantly increased (*p* < 0.05) (Figure 1D), and the activities of SOD and GSH-Px were significantly decreased (*p* < 0.05) (Figure 1E,F), indicating that the lipid peroxide content of liver of the high-lipid mice was significantly increased, and the free radical scavenging ability was significantly decreased. The overall oxidative stress level of the mice was increased due to high fat. LAB intervention resulted in significant improvements in hepatic oxidative status, characterized by decreased MDA concentration (*p* < 0.05, Figure 1D) and enhanced SOD and GSH-Px activities (*p* < 0.05, Figure 1E,F). Although SOD activity was restored to control levels, the GSH-Px activity and MDA content showed persistent alterations compared to the control group (*p* < 0.05). Khanna et al. obtained similar results, demonstrating that probiotics *Lactiplantibacillus pentosus* GSSK2, *Limosilactobacillus fermentum* PUM, and *Lactiplantibacillus plantarum* GS26A significantly reduced MDA levels in the livers of HFD-fed rats and significantly increased SOD and GSH levels [19]. These data suggest that LAB exerts hepatoprotective effects by modulating the anti-oxidant defense system and reducing oxidative damage.

The liver is the main place of lipid metabolism in the body, so the disorder of lipid metabolism caused by a high-fat diet will cause abnormal lipid metabolism in the liver, which will lead to excessive lipid accumulation in the liver, and eventually lead to deformation of liver cells and large-scale fat accumulation degeneration [10]. Figure 1G shows the HE staining results of the paraffin sections of the mice livers in each group. It can be seen that the liver tissues of the mice in the control group are dense, and the liver cells are arranged regularly without obvious fat particles. However, in the HFD group, the density of liver tissue decreased, the arrangement of hepatocytes was disordered, and there were many fat particles with high density, which indicated that feeding with high-fat feed had a significant effect on the liver of the mice, and the obesity model was successfully induced, which was consistent with the result that the wet weight of liver increased significantly. Compared with the HFD group, the liver tissues and hepatocytes in the HFD+LL group and HFD+LP group are arranged normally, and the fat particles are significantly reduced, which shows that the intragastric administration of LAB can significantly reduce the fat content and reduce the wet weight of liver in hyperlipidemia mice. In addition, there is no significant difference in the distribution of liver tissue and fat particles between the C+LL and C+LP groups and the control group, which shows that LAB has no significant effect on the liver tissue of normal mice. The abnormal accumulation of fat will cause liver cell damage and dysfunction, which further explains the abnormal increase in serum ALT and AST levels in mice fed with a high-fat diet.

### 3.4. Production of SCFAs

SCFAs, comprising acetic acid, propionic acid, isobutyric acid, butyric acid, isovaleric acid, and valeric acid, serve as crucial microbial metabolites that regulate colonic epithelial integrity and maintain large intestinal homeostasis [20]. An analysis of SCFA profiles revealed significant alterations in the HFD-fed mice compared to the controls, with marked reductions in propionic acid, isobutyric acid, butyric acid, isovaleric acid, and valeric acid concentrations (*p* < 0.05, Table 2), indicating the HFD-induced disruption of microbial SCFA production. Notably, LAB supplementation (HFD+LL and HFD+LP groups) effectively restored SCFA levels to those comparable with control animals, demonstrating the therapeutic potential of LAB in ameliorating HFD-induced SCFA dysregulation. Studies have found that in addition to providing energy to the host, SCFAs can act as an important regulatory factor regulating lipid metabolism [21]. SCFAs can be activated by G protein-coupled receptors (GPCRs) and peroxisome proliferators-activated receptor γ, PPARγ, which regulates energy expenditure and insulin sensitivity in surrounding metabolic tissues to affect the development of obesity. Some studies have found that supplementation with dietary SCFAs supplementation can activate the expression of GPR41 to improve liver metabolic function, thereby inhibiting liver mass increase and lipid synthesis, and delaying the occurrence of obesity induced by high-fat diet in mice [22]. Acetic acid can activate the G (i/o) β-γ-phospholipase C-protein kinase C signaling pathway mediated by GPR43; up-regulate the expression of tumor suppressant genes; inhibit the phosphorylation of protein kinase B, improve systemic insulin sensitivity; improve fat accumulation in white adipose tissue; regulate lipid and glucose metabolism in skeletal muscle, liver, and other organs; and improve energy utilization to delay obesity [23]. Acetic acid can also promote lipid oxidation through the AMPK/PGC-1α/PPARα pathway [24]. Propionate can activate GPR41 signaling and promote the expression of the Ca^2+^-mediated insulin secretion I/O sensitive pathway, thereby increasing body energy consumption [25]. Propionic acid can also activate GPR109A activation in adipose tissue, stimulate lipase activity, and reduce plasma triglyceride and free fatty acid levels. Butyric acid can modulate GPR41 and GPR43 to up-regulate the expression of calcium/calmodulin-dependent protein kinase II in hepatocytes, inhibit the expression of histone deacetylase 1 in Hep1-6 cells, and activate the phosphorylation of transcription enhancer cyclic adenosine phosphate-effecting-element binding protein to improve liver steatosis, lipid metabolism abnormalities, and delay the occurrence of obesity. Li et al. conducted a 9-week comparative experimental intervention between high-fat diets with and without butyrate and found that butyrate supplementation could reduce the neuronal activity of neuropeptide Y’s appetite neurons, the nucleus tractus solitarius of the brainstem, and the dorsal vagus nerve complex, thus inhibiting appetite [26]. They also found that butyrate can promote fatty acid oxidation and activate brown adipose tissue, reduce energy intake, and delay the occurrence of obesity. These findings may explain how LAB can significantly change SCFA levels and delay obesity and hyperlipidemia induced by high-fat diet.

### 3.5. Effect of LAB on Gut Bacterial Diversity Indices in Mice

**Figure 2:**
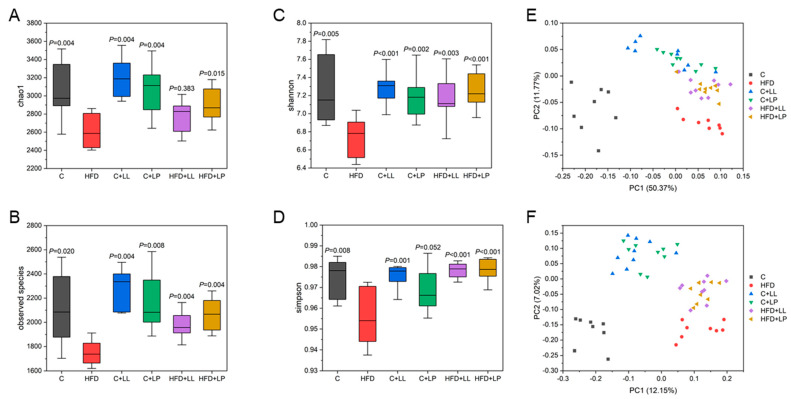
Effects of LAB on the alpha-diversity index and beta-diversity index of the gut microbiota in mice: (**A**) chao1 index; (**B**) observed species; (**C**) shannon index; (**D**) simpson index; (**E**) PCoA plot based on weighted UniFrac distance; (**F**) PCoA plot based on unweighted UniFrac distance. Compared with the HFD group, the difference was statistically significant at *p* < 0.05.

Microbial α-diversity analysis was conducted at a 97% similarity threshold, with the results presented in Figure 2A–D. Four key indices were employed to assess community diversity: the observed species index, which estimates total species richness within the microbial community; the chao1 index, reflecting the presence of low-abundance species; the Shannon index, quantifying community diversity and uncertainty; and the Simpson index, measuring species dominance and evenness [27]. Compared with the control group, the α-diversity index of the HFD group was significantly decreased (*p* < 0.05), indicating that a high-fat diet significantly reduced the overall species abundance, rare species abundance, and distribution uniformity of gut microbiota in mice. All the LAB-treated groups demonstrated significant improvements in the α-diversity indices compared to the HFD group (*p* < 0.05), reaching levels comparable to the control group. These findings suggested that the LAB intervention effectively restored gut microbial diversity and species richness in the HFD-fed mice.

The β-diversity analysis was performed to compare microbial community composition across different samples. Principal coordinates analysis (PCoA) based on both weighted and unweighted Unifrac distances revealed distinct clustering patterns (Figure 2E,F). The weighted Unifrac analysis accounted for 50.37% and 11.77% of variation along the first two principal coordinates, while unweighted Unifrac explained 12.15% and 7.02% of the variance. Distinct separation and scattered distribution patterns were observed between the control and HFD groups, reflecting substantial inter-individual variability. In contrast, the LAB-treated groups demonstrated tighter clustering with reduced dispersion, indicating more consistent microbial profiles and suggesting that both LAB formulations effectively modulated gut microbiota composition with greater uniformity.

### 3.6. Effect of LAB on the Gut Bacterial Community Structure in Mice

#### 3.6.1. Species Distribution and Relative Abundance Differences at the Phylum Level

**Figure 3:**
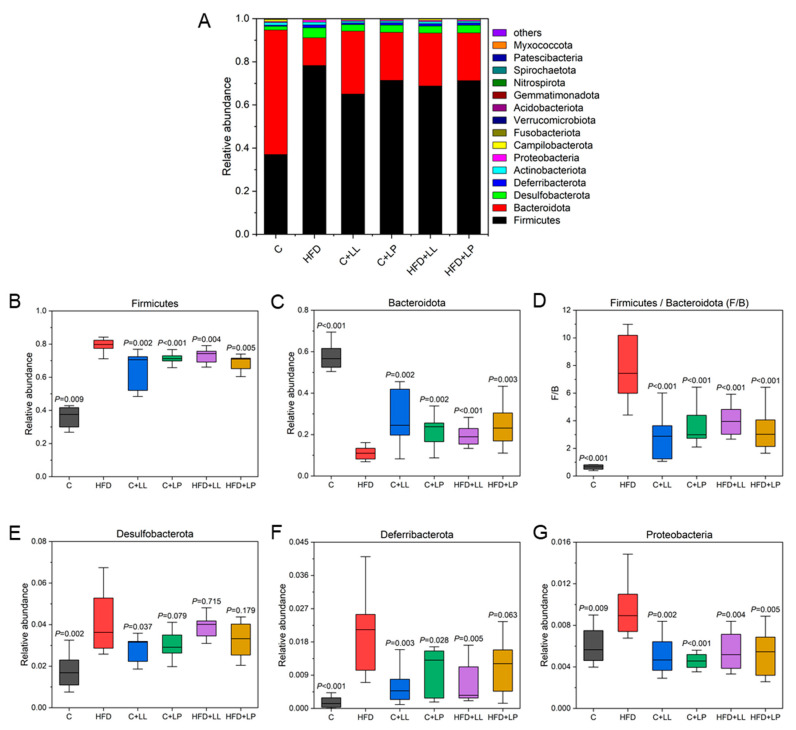
Effects of LAB on the distribution and relative abundance of the gut microbiota in mice at the phylum level. (**A**) Overall distribution of the first 15 phyla; (**B**) *Firmicutes*; (**C**) *Bacteroidetes*; (**D**) *Firmicutes*/*Bacteroidetes* ratio; (**E**) *Desulfobacterota*; (**F**) *Deferribacteres*; (**G**) *Proteobacteria*. Compared with the HFD group, the difference was statistically significant at *p* < 0.05.

Using the Vsearch software (V2.29.0, https://github.com/torognes/vsearch/releases/tag/v2.29.0 (accessed on 19 April 2025)), sequence data from all 60 samples were clustered into operational taxonomic units (OTUs) at a 97% similarity threshold. Taxonomic annotation of the OTU sequences enabled classification and relative abundance determination across multiple taxonomic levels (phylum, class, order, family, and genus). At the phylum level, *Firmicutes* and *Bacteroidetes* dominated the gut microbiota, collectively representing >90% of the microbial community in all the experimental groups (Figure 3A). The relative abundances of *Desulfobacterota*, *Deferribacterota*, and *Actinobacteriota* ranged from 1% to 5%, while the relative abundances of other bacteria, such as *Proteobacteria*, *Campilobacterota*, *Fusobacteriota*, *Verrucomicrobiota*, *Acidobacteriota*, *Gemmatimonadota*, *Nitrospirota*, *Spirochaetota*, *Patescibacteria*, and *Myxococcota*, were less than 1%.

The high-fat diet (HFD) significantly altered the gut microbial composition, increasing the relative abundance of *Firmicutes* (*p* < 0.05, Figure 3B) while decreasing *Bacteroidetes* (*p* < 0.05, Figure 3C) compared to the controls. The LAB intervention modulated these changes, significantly reducing *Firmicutes* and increasing *Bacteroidetes* compared to the HFD group, though levels remained distinct from the controls (*p* < 0.05). As key regulators of energy homeostasis, *Firmicutes* and *Bacteroidetes* play crucial roles in nutrient absorption, energy conversion, and glucose metabolism [28]. The *Firmicutes*/*Bacteroidetes* (F/B) ratio, a recognized indicator of metabolic health, is positively correlated with enhanced caloric extraction from diet and associated with obesity and type 2 diabetes development [2], while reduced F/B ratios are characteristic of lean phenotypes. These phyla encode numerous carbohydrate-active enzymes that facilitate indigestible polysaccharide utilization [29], with Bacteroidetes particularly contributing to short-chain fatty acid production through specialized glycoside hydrolases and metabolic pathways [28]. Our findings suggest that LAB may regulate host energy balance and lipid metabolism by modulating the F/B ratio (Figure 3D). Furthermore, the LAB treatment significantly reduced the relative abundance of potentially pathogenic phyla, including *Desulfobacterota*, *Deferribacteres*, and *Proteobacteria*, compared to the HFD group (*p* < 0.05, Figure 3E–G). These reductions may contribute to improved gut health, as *Desulfobacterota* can release pro-inflammatory lipopolysaccharides, *Deferribacteres* expansion has been linked to inflammatory bowel disease, and *Proteobacteria* encompasses numerous pathogenic genera such as *Escherichia*, *Salmonella*, and *Helicobacter* [30]. The LAB-mediated suppression of these phyla suggests a protective effect against HFD-induced dysbiosis and associated metabolic disturbances.

#### 3.6.2. Representative Species Difference at the Genus Level

**Figure 4:**
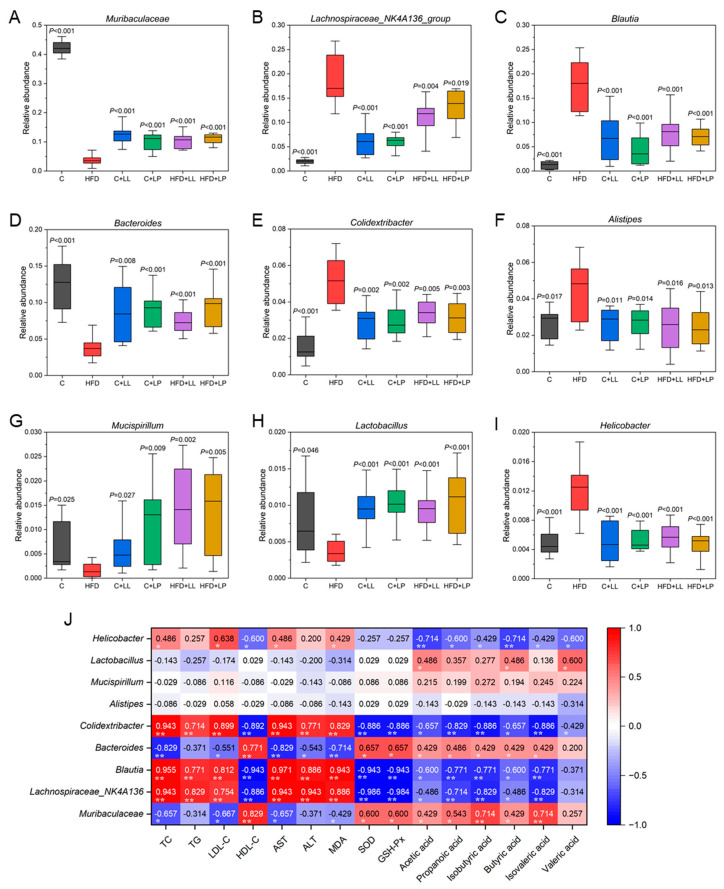
Effects of LAB on the relative abundances of main species in the gut microbiota of the mice at the genus level (**A**–**I**), and Spearman’s correlation coefficients between serum lipids, liver oxidative stress levels, gut short-chain fatty acids (SCFAs), and representative genus of the gut microbiota in the mice (**J**). Compared with the HFD group, the difference was statistically significant at *p* < 0.05 (**A**–**I**). * *p* < 0.05; ** *p* < 0.01 (**J**).

High-fat diet (HFD) feeding resulted in significant reductions in the relative abundance of key genera, including *Muribaculaceae*, *Bacteroides*, *Mucispirillum*, and *Lactobacillus*. However, the LAB intervention effectively restored these microbial populations to levels comparable with the control group (Figure 4A,D,G,H). A high-fat diet significantly increased the relative abundances of *Lachnospiraceae*_NK4A136_group, *Blautia*, *Colidextribacter*, *Alistipes*, and *Helicobacter* in the gut of the mice; however, after the LAB treatment, the abundances fell back to the control level (Figure 4B,C,E,F,I). One study showed that mice with high cholesterol had the highest *Muribaculaceae* in their guts after ingesting *Lactiplantibacillus plantarum* H6, indicating that *Muribaculaceae* can act as a potential biomarker for effective cholesterol reduction [31]. *Mucispirillum* was reported as a health marker of the colon mucosa, showing a decline in abundance during early infection with pathogenic microorganisms and a return to normal abundance during the recovery period (after pathogen removal). *Bacteroidetes* and *Lactobacillus* in the gut microbiota participate in the hydrolysis of polysaccharides into SCFAs. *Lactobacillus* can effectively scavenge oxidative stress levels through lactate oxidase, NADH oxidase, superoxide dismutase, and pyruvate oxidase, thereby alleviating oxidative stress levels [32]. Bacteria such as *Lachnospiraceae* from *Firmicutes* may contribute to obesity and type 2 diabetes. Duan et al. found that flavonoids from whole-grain oats can significantly increase *Akkermansia* and significantly decrease *Lachnoclostridium*, *Blautia*, *Colidextribacter*, and *Desulfovibrio*, and these bacteria were strongly correlated with hyperlipidemia-related indicators according to Spearman’s correlation analysis [33]. The obtained results suggest that LAB may increase the level of short-chain fatty acids in the gut tract by increasing the content of beneficial bacteria such as *Muribaculaceae*, *Bacteroides*, *Mucispirillum*, and *Lactobacillus* and reducing the content of *Lachnospiraceae*, *Blautia*, *Colidextribacter*, *Alistipes*, and *Helicobacter* and other pathogenic bacteria to regulate the gut microbiota of mice and affect the carbohydrate metabolism, energy absorption, and immunity of the host.

### 3.7. Correlations Between the Serum Lipids, Liver Oxidative Stress Levels, Gut SCFAs, and Bacteria Taxa

The relationships between the serum lipids, liver oxidative stress levels, gut SCFAs, and bacteria taxa were analyzed using Spearman’s rank correlation, with the results presented in Figure 4J. In terms of lipid levels in the mice serum, *Lachnospiraceae*_NK4A136, *Blautia*, and *Colidextribacter* showed extremely significant positive correlations (*p* < 0.01) with TC, TG, LDL-C, AST, and ALT, while they exhibited extremely significant negative correlations (*p* < 0.01) with HDL-C. Conversely, *Muribaculaceae*, *Bacteroides*, and *Lactobacillus* demonstrated negative correlations with TC, TG, LDL-C, AST, and ALT, and positive correlations with HDL-C. Regarding oxidative stress levels in mice livers, MDA showed extremely significant positive correlations (*p* < 0.01) with *Lachnospiraceae*_NK4A136, *Blautia*, and *Colidextribacter*, and a significant positive correlation (*p* < 0.05) with *Helicobacter*. In contrast, SOD and GSH-Px exhibited opposite trends. Specifically, *Muribaculaceae*, *Bacteroides*, and *Lactobacillus* showed negative correlations with MDA levels and positive correlations with SOD and GSH-Px. These results indicate that *L. lactis* subsp. *lactis* LL-1 and *L. paracasei* LP-16 influence mice lipid levels and liver oxidative stress by regulating gut microbiota structure. This effect is closely related to whether the microbiota is beneficial or harmful bacteria, as the beneficial bacteria are typically associated with low lipid and oxidative stress levels [31], while harmful bacteria often lead to elevated lipid and oxidative stress levels [33]. Pan et al. found that *Limosilactobacillus fermentum* 016 can enhance the hepatic anti-oxidant indices and intestinal mucosal barrier function in mice. Furthermore, *L. fermentum* 016 significantly increases the abundance of beneficial bacterial genera and elevates tryptophan metabolite levels, thereby reversing metabolic disorders [34]. Therefore, the “gut–liver axis” plays a pivotal role in the pathogenesis of metabolic liver diseases, as alterations in the gut microbiota can influence hepatic oxidative stress levels through multiple pathways [35].

Positive correlations were observed between SCFA levels and the abundance of *Muribaculaceae*, *Bacteroides*, *Mucispirillum*, and *Lactobacillus*, while negative correlations were found with *Lachnospiraceae*, *Blautia*, *Colidextribacter*, *Alistipes*, and *Helicobacter*. Specifically, acetic acid showed significant positive correlations with *Muribaculaceae* and *Bacteroides* (*p* < 0.05), and a highly significant positive correlation with *Lactobacillus* (*p* < 0.01). Propanoic acid was extremely significantly positively correlated with the enrichment of *Muribaculaceae*, *Bacteroides*, and *Lactobacillus* (*p* < 0.01). Butyric acid was significantly positively correlated with the enrichment of *Muribaculaceae* and *Lactobacillus* (*p* < 0.05) and extremely significantly positively correlated with the enrichment of *Bacteroides* (*p* < 0.01). SCFAS are mainly undigested dietary fiber produced by anaerobic bacterial fermentation in the cecum and large intestine. The predominant SCFA production profiles differ between major bacterial phyla, with *Bacteroides* (Gram-negative) primarily generating acetate and propionate, while *Firmicutes* (Gram-positive) predominantly synthesize butyrate. Based on the assessment of factors such as diet, type and amount of microbiome, and residence time in the intestine, the gut produces 500–600 mmol of SCFAs per day [36]. *Bacteroidetes*, *Bifidobacterium*, *Eubacterium*, *Ruminococcus*, *Peptostreptococcus*, and *Clostridium* are the main groups of acetic acid-producing bacteria; *Clostridium* is the main propionic acid-producing bacteria; and *Bacteroides*, *Eubacillus*, and *Clostridium* are the main bacteria that produce butyric acid. The results of this study indicate that LAB enhanced the production of SCFAs by enriching the gut flora that produced SCFAs to improve the symptoms of obesity and hyperlipidemia in mice induced by high-fat diets.

### 3.8. Multivariate Statistical Analysis of Gut Metabolites in Mice

**Figure 5:**
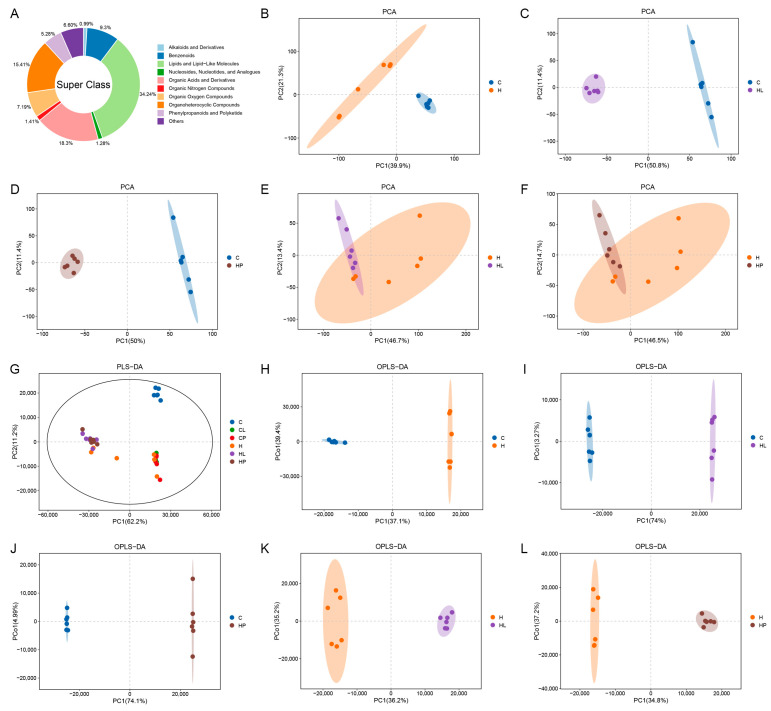
Multivariate statistical analysis results of the gut metabolites in the mice: (**A**) Classification pie chart of gut metabolites in mice. (**B**–**F**) PCA diagram of the gut metabolites in the mice. (**G**) PLS-DA plot of the gut metabolites in the mice. (**H**–**L**) PLS-DA plot of the gut metabolites in the mice.

A total of 9393 metabolites were detected in the gut content samples of all the mice, and Figure 5A shows the pie classification chart. The SuperClass classification system categorizes murine gut metabolites into distinct chemical classes, including alkaloids, aromatic compounds, lipids, nucleosides, etc. Among them, lipid and lipid molecules (34.24%), organic acids and their derivatives (18.30%), organic heterocyclic compounds (15.41%), and 6.61% of metabolites were not clearly classified.

The PCA analysis results of the gut metabolites of the mice in each group are shown in Figure 5B–F. The results showed that the distance between the individual samples of the 6 groups was close, while the distance between the groups of the control group (C), high-fat group (H), high-fat with LL group (HL), and high-fat with LP group (HP) was far from each other, and there was no intersection, indicating that the gut metabolites of the mice in the high-fat group and all the lactic acid bacteria groups had significant changes. The distance between the H group and HL and HP groups overlaps, but there is still a certain distance, indicating that the gut metabolites of hyperlipidemia mice still have certain changes after lactic acid bacteria administration, and lactic acid bacteria may play a certain role in regulating the gut metabolites of the mice. The PLS-DA analysis results of the mouse gut metabolites are shown in Figure 5G. The two principal components explain the variance of 62.2% and 11.2%, respectively, and the cumulative contribution rate is greater than 70%, which indicates that the model is good. The results of the PLS-DA analysis are consistent with those of the PCA analysis. The results of the OPLS-DA analysis of the mouse gut metabolites are shown in Figure 5H–L. The distance between group C and groups H, HL, and HP, and the distance between groups H, HL, and HP were far and had no intersection, indicating that the gut metabolites of the mice in the high-fat group and all the groups of LAB administration had significant changes, and the gut metabolites of the mice with hyperlipidemia also had significant changes after the lactic acid bacteria administration. The results of the OPLS-DA analysis further showed that lactic acid bacteria played a significant role in regulating the gut metabolites in the mice.

### 3.9. Differential Metabolites of Gut Metabolites in Mice

**Figure 6:**
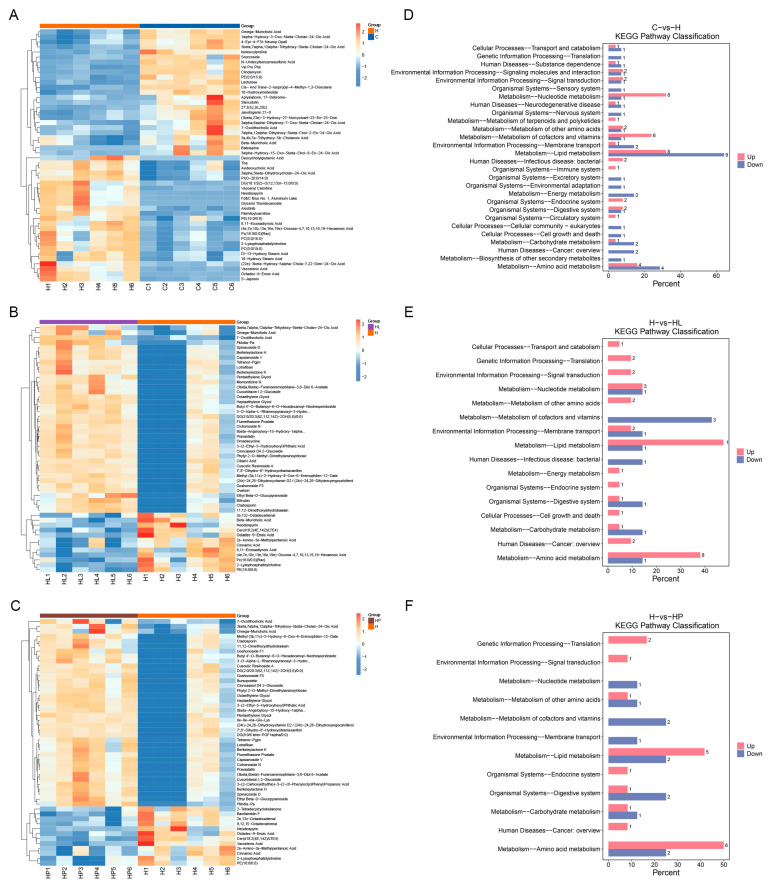
Analysis of differential gut metabolites (**A**–**C**) and KEGG pathway enrichment (**D**–**F**) in mice.

Multivariate and univariate analytical approaches were employed to identify group-specific differential metabolites. In both the OPLS-DA and PLS-DA models, the variable importance in projection (VIP) score served as a quantitative measure of each metabolite’s contribution to group discrimination and classification accuracy. This approach enabled the identification of biologically relevant differential metabolites, which were subsequently validated for statistical significance using independent *t*-tests. The clustering heat maps of different gut metabolites of mice in each group are shown in Figure 6A–C. The results showed that compared with the control group (C), the gut deoxycholic acid, aboxycholic acid, 3α, and 3α of mice in the high-fat group (H) were significantly higher than those in the control group (C). The relative abundance of 5β-dihydroxycholic acid-24-oleic acid, glycerin undecanoate, palmitoyl carnitine, 8,11-eicosadienoic acid, docosatetraenoic acid, 2-lysophosphatidylcholine, 13-hydroxystearic acid, 16-hydroxystearic acid, (22e)-3β-hydroxyl-5α-choline-7,22-dien-24-oleic acid, octadecane-9-ene acids was significantly up-regulated, indicating that the high-fat diet mainly caused an increase in the content of bile acids and long-chain fatty acids in the intestines of the mice.

Research has demonstrated that the majority of bile acids undergo enterohepatic circulation, being reabsorbed in the ileum and transported to the liver through the portal vein, with only a minor fraction reaching the large intestine. The regulatory effects of bile acids on lipid metabolism vary according to their specific chemical structures. The gut microbiota plays a crucial role in lipid homeostasis by modulating the size and composition of the bile acid pool. Hyperlipidemic conditions are characterized by increased total bile acid synthesis, expansion of the bile acid pool, and significant alterations in the ratio of primary to secondary bile acids. Furthermore, saturated fatty acids prevalent in high-fat diets have been shown to stimulate bile acid secretion, thereby modifying the intestinal microbial environment [37]. Abnormal choline metabolism is a hallmark of tumorigenesis and cancer progression, characterized by increased levels of choline phosphate, glycerophosphate choline, and total choline compounds. In the choline cycle, membrane phosphatidylcholine is synthesized from free choline via a carcinogenic signaling pathway, where free choline is phosphorylated to choline phosphate with the help of choline kinase, and then converted to cytidine diphosphate choline by adding a cytidine diphosphate group to choline phosphate. In the next step, diacylglycerol (DAG)-choline phosphotransferase uses diacylglycerol as a lipid to catalyze the final reaction and produces membrane phosphatidylcholine with cytidine diphosphate-choline. Therefore, the results of this study also showed that the increased contents of long-chain fatty acids, bile acids, and phosphatidylcholine in the gut of the mice in the high-fat group, as well as the structural changes in the gut flora described above, promoted the growth of some potential pathogens in the gut and adversely affected the lipid metabolism of the host.

Compared with the high-fat group (H), the relative abundance of bile acids such as deoxycholic acid, aboxycholic acid, and rhombocholic acid, as well as long-chain fatty acid metabolites such as octadecadienoic acid, octadecadienoic acid, cinnamic acid, 8,11−eicosadienoic acid, and docosatetraenoic acid in the gut tract of the mice in the lactic acid bacteria group (HL and HP), were significantly reduced. The relative abundances of 7-oxalic acid, glucoside, octoethylene glycol, heptanediol, pentaglycol, Ile-Ile-Ala-Glu-Lys, and dihydroxyvitamin D2 were significantly up-regulated. In conclusion, hyperlipidemia mice can reduce the contents of bile acid and long-chain fatty acids in the intestine by regulating the gut flora after the administration of lactic acid bacteria while increasing the contents of short-chain fatty acids, short peptides, vitamins, and other substances, regulate gut metabolites, improve gut metabolite disorders caused by hyperlipidemia, and promote gut health of the host.

Numerous studies have identified vitamin D deficiency as a common comorbidity in obesity, with higher prevalence rates among obese individuals [38]. An inverse relationship has been observed between serum vitamin D levels and BMI, with weight reduction leading to improved vitamin D status. Clinical intervention studies have confirmed that vitamin D supplementation significantly reduces obesity-related parameters, including body weight, BMI, and waist–hip circumference. Vitamin D deficiency may also lead to dyslipidemia, a disorder often associated with impaired glucose homeostasis [39]. Studies have found that vitamin D supplementation can reduce the levels of TC, LDL, and TG in the blood [40]. Moreover, a study revealed that LDL and TG levels exhibited an inverse relationship with serum vitamin D concentrations, whereas reduced HDL levels were linked to lower serum vitamin D levels [41]. In view of these conditions, it has been proposed that vitamin D can be used clinically as an adjunct therapy for statins in the treatment of hypercholesterolemia [42], but the exact mechanism of action is still unclear. The results indicated that vitamin D2 metabolites in the gut of mice increased after the administration of probiotic lactic acid bacteria powder HL and HP, indicating that lactic acid bacteria may synthesize vitamin D2 in the gut, and then reduce the levels of serum TC, TG, and LDL, which is consistent with the results of the study on lowering blood lipids (Table 1).

### 3.10. KEGG Pathway Enrichment Analysis of Differential Metabolites in Mice Gut

Pathway enrichment analysis of differential metabolites provides valuable insights into the underlying mechanisms of metabolic pathway alterations in comparative samples. Figure 6D–F illustrates the KEGG level 2 classification of both the up-regulated and down-regulated metabolites across the different experimental groups in the murine gut. The results showed that compared with the group C and H, the gut pathways related to lipid metabolism, nucleotide metabolism, and amino acid metabolism were more enriched, and the relative contents of up-regulated and down-regulated metabolites were significantly different. The relative contents of up-regulated and down-regulated metabolites related to lipid metabolism were 32.00% and 64.29%, respectively. The relative contents of up-regulated and down-regulated metabolites related to nucleotide metabolism were 32.00% and 7.14%, respectively. The relative contents of up-regulated and down-regulated metabolites related to amino acid metabolism were 16.00% and 28.57%, respectively. It was shown that a high-fat diet led to an increase in the metabolites associated with lipid metabolism in the gut of the mice, which was associated with an increase in the content of lipid-associated long-chain fatty acids in the gut differential metabolite analysis results.

Compared with HL, the enrichment of the pathways related to lipid metabolism, coenzyme factor, vitamin metabolism, and amino acid metabolism in the gut of the mice in group H was relatively large, and the relative contents of the up-regulated and down-regulated metabolites were significantly different. The relative contents of the up-regulated and down-regulated metabolites related to lipid metabolism were 47.62% and 14.29%, respectively. The relative contents of up-regulated and down-regulated metabolites related to coenzyme factor and vitamin metabolism were 0.00% and 42.86%, respectively. The relative contents of the up-regulated and down-regulated metabolites related to amino acid metabolism were 38.10% and 14.29%, respectively. The results showed that *L. lactis* subsp. *lactis* LL-1 induced the decrease in metabolites related to lipid metabolism in the gut tract of the hyperlipidemia mice, which was related to the decrease in long-chain fatty acid content in the gut differential metabolite analysis. Moreover, the proportion of metabolites related to coenzyme factors and vitamin metabolism increased, which was related to the increase in vitamin content in the gut differential metabolite analysis results, which was conducive to improving hyperlipidemia caused by gut metabolite disorders and promoting host gut health.

Compared between group H and HP, the results were similar to those of group HL, and the gut pathways related to lipid metabolism, coenzyme factor metabolism, vitamin metabolism, and amino acid metabolism were relatively enriched, and the relative contents of up-regulated and down-regulated metabolites were significantly different. The relative contents of up-regulated and down-regulated metabolites related to lipid metabolism were 41.67% and 25.00%, respectively. The relative contents of up-regulated and down-regulated metabolites related to coenzyme factor and vitamin metabolism were 0.00% and 25.00%, respectively. The relative contents of up-regulated and down-regulated metabolites related to amino acid metabolism were 50.00% and 25.00%, respectively. The results showed that *L. paracasei* LP-16 has the same regulation effect on gut metabolites as *L. lactis* subsp. *lactis* LL-1.

## 4. Conclusions

LAB (*L. lactis* subsp. *lactis* LL-1 and *L. paracasei* LP-16) significantly reduced obesity, lipid levels, and oxidative stress levels in mice induced by a high-fat diet. A high-fat diet significantly reduced the overall species abundance, rare species abundance, and distribution uniformity of the gut microbiota in mice, while treatment with LAB increased the species diversity of the gut microbiota, with a more uniform distribution. At the phylum level, LAB may help mitigate excessive energy absorption from the diet and regulate body weight by modulating the relative proportions of *Firmicutes* and *Bacteroidetes*. Additionally, LAB can suppress the proliferation of certain harmful bacteria (such as *Desulfobacterota*, *Deferribacteres*, and *Proteobacteria*) in the gut of mice induced by a high-fat diet. At the genus level, MDA showed extremely significant positive correlations (*p* < 0.01) with *Lachnospiraceae*_NK4A136, *Blautia*, and *Colidextribacter*, and a significant positive correlation (*p* < 0.05) with *Helicobacter*, while SOD and GSH-Px exhibited opposite trends. Specifically, *Muribaculaceae*, *Bacteroides*, and *Lactobacillus* showed negative correlations with MDA levels and positive correlations with SOD and GSH-Px. The levels of short-chain fatty acids (SCFAs) showed a positive association with the abundance of *Muribaculaceae*, *Bacteroides*, *Mucispirillum*, and *Lactobacillus* while exhibiting a negative correlation with the abundance of *Lachnospiraceae*, *Blautia*, *Colidextribacter*, *Alistipes*, and *Helicobacter*. LAB increased the SCFA level in the gut by increasing the content of beneficial bacteria and reducing the content of pathogenic bacteria to improve the symptoms of obesity and hyperlipidemia in mice. According to the results of this article, there is no significant difference between *L. lactis* subsp. *lactis* LL-1 and *L. paracasei* LP-16 in the function of blood lipid reduction and regulating the gut microbiota. The results of the gut differential metabolite analysis showed that the high-fat group of mice had increased levels of long-chain fatty acids and bile acids in the intestine, as well as changes in the gut flora structure mentioned above, which promoted the growth of some potential pathogens in the intestine and had an adverse effect on the host’s lipid metabolism. The intragastric administration of *L*. *lactis* subsp. *lactis* LL-1 and *L. paracasei* LP-16 can lead to a decrease in metabolites related to lipid metabolism in the intestines of hyperlipidemic mice, which is consistent with the results of the gut differential metabolite analysis. The content of lipid-related long-chain fatty acids is reduced, and the proportion of metabolites related to coenzyme factors and vitamin metabolism is increased. This is related to the increase in vitamin content in the gut differential metabolite analysis results, which is beneficial to improving the gut symptoms caused by hyperlipidemia. Our results provide a theoretical basis for the use of *L. lactis* subsp. *lactis* LL-1 and *L. paracasei* LP-16 as potential functional probiotics for weight loss and blood lipid reduction.

In the design of animal experiments, the inclusion of a positive control group, such as the oral administration of chemical lipid-lowering agents (statins), would enable a comparison of the differences in lipid-lowering efficacy between the two LAB strains and chemical drugs. Additionally, methodologies conducive to transcriptomic studies can identify gene expression differences in LAB that regulate oxidative stress levels and are associated with lipid metabolism, as well as their correlation with the gut microbiota, thereby elucidating the mechanisms by which LAB lowers blood lipid levels. Relevant research is currently underway. Furthermore, there exist fundamental differences between animals and humans, and the efficacy of drugs in animals cannot be directly equated to their effects in humans. Different animal species possess distinct physiological functions and metabolic characteristics. Therefore, to ensure the validity of the experimental results, comparative observations should be conducted using multiple animal models, such as rats, rabbits, dogs, and monkeys. Through animal experiments involving various dosages and administration routes, the most effective dosage range and optimal delivery method can be identified, providing references for subsequent human trials. Both LAB strains evaluated in this study are edible and could be further assessed for their clinical lipid-lowering effects through human trials at an appropriate time in the future.

## Figures and Tables

**Table 1:** Effects of LAB on serum biochemical indices in mice.

Group	TC (mmol/L)	*p*-Value	TG (mmol/L)	*p*-Value	LDL-C (mmol/L)	*p*-Value	HDL-C (mmol/L)	*p*-Value	AST (U/L)	*p*-Value	ALT (U/L)	*p*-Value
C	4.10 ± 0.34	0.003	0.78 ± 0.10	0.001	0.46 ± 0.03	<0.001	5.08 ± 0.23	<0.001	141.87 ± 8.64	0.003	40.21 ± 4.25	<0.001
HFD	5.78 ± 0.29	-	1.49 ± 0.12	-	0.88 ± 0.03	-	2.69 ± 0.18	-	188.90 ± 8.58	-	110.49 ± 6.38	-
C+LL	4.15 ± 0.26	0.002	0.77 ± 0.08	<0.001	0.42 ± 0.04	<0.001	4.99 ± 0.25	<0.001	146.21 ± 8.41	0.004	41.27 ± 3.97	<0.001
C+LP	4.13 ± 0.25	0.002	0.76 ± 0.06	<0.001	0.46 ± 0.03	<0.001	4.87 ± 0.23	<0.001	142.62 ± 8.06	0.002	39.92 ± 4.06	<0.001
HFD+LL	4.73 ± 0.32	0.014	0.95 ± 0.09	0.003	0.51 ± 0.03	<0.001	4.73 ± 0.20	<0.001	154.10 ± 8.85	0.009	70.91 ± 5.66	0.001
HFD+LP	4.66 ± 0.30	0.010	0.99 ± 0.10	0.005	0.49 ± 0.04	<0.001	4.80 ± 0.26	<0.001	151.55 ± 8.62	0.006	71.98 ± 5.89	0.002

Compared with the HFD group, the difference was statistically significant at *p* < 0.05.

**Table 2:** Concentrations of short-chain fatty acids (SCFAs) in mice with different treatments.

Group	SCFAs (mmol/L)											
	Acetic Acid	*p*-Value	Propanoic Acid	*p*-Value	Isobutyric Acid	*p*-Value	Butyric Acid	*p*-Value	Isovaleric Acid	*p*-Value	Valeric Acid	*p*-Value
C	10.46 ± 0.41	0.402	7.91 ± 0.22	<0.001	1.36 ± 0.04	<0.001	8.05 ± 0.31	0.011	1.78 ± 0.06	<0.001	1.25 ± 0.04	0.007
HFD	10.15 ± 0.40	-	4.95 ± 0.24	-	0.98 ± 0.04	-	6.90 ± 0.32	-	0.99 ± 0.05	-	1.04 ± 0.06	-
C+LL	15.18 ± 0.67	<0.001	7.60 ± 0.38	<0.001	1.32 ± 0.05	<0.001	9.90 ± 0.50	<0.001	1.62 ± 0.08	<0.001	1.72 ± 0.07	<0.001
C+LP	13.53 ± 0.50	<0.001	6.81 ± 0.28	<0.001	1.16 ± 0.03	0.003	8.72 ± 0.32	0.002	1.32 ± 0.05	0.001	1.12 ± 0.04	0.127
HFD+LL	10.43 ± 0.22	0.348	5.67 ± 0.14	0.011	1.29 ± 0.03	<0.001	7.31 ± 0.23	0.146	1.40 ± 0.05	<0.001	1.19 ± 0.05	0.029
HFD+LP	11.26 ± 0.40	0.027	5.94 ± 0.20	0.005	1.19 ± 0.04	0.003	8.00 ± 0.30	0.012	1.21 ± 0.06	0.008	1.08 ± 0.06	0.460

Compared with the HFD group, the difference was statistically significant at *p* < 0.05.

## Data Availability

All the data are contained within the article, and the data presented in this study are available upon request from the corresponding author.
